# Lung Ultrasound Versus Chest Radiography for Acute Heart Failure: Impact of Heart Failure History and Pleural Effusion

**DOI:** 10.3390/diagnostics15233047

**Published:** 2025-11-28

**Authors:** Kristina Cecilia Miger, Anne Sophie Overgaard Olesen, Johannes Grand, Mikael Ploug Boesen, Jens Jakob Thune, Olav Wendelboe Nielsen

**Affiliations:** 1 Department of Cardiology, Copenhagen University Hospital–Bispebjerg and Frederiksberg, 2200 Copenhagen, Denmark; 2 Department of Clinical Medicine, University of Copenhagen, 2200 Copenhagen, Denmark; 3 Department of Cardiology, Copenhagen University Hospital–Amager and Hvidovre, 2650 Copenhagen, Denmark; 4 Department of Radiology, Copenhagen University Hospital–Bispebjerg and Frederiksberg, 2200 Copenhagen, Denmark

**Keywords:** acute heart failure, emergency department, lung ultrasound, chest radiographs, chest computed tomography, pulmonary congestion

## Abstract

**Background/Objectives:** This is the first prospective, same-day, multi-modality comparison of lung ultrasound (LUS) and chest radiography (CXR) for detecting acute heart failure (AHF) in non-critical patients with dyspnoea, examining the impact of chronic heart failure and pleural effusion, using low-dose chest CT (LDCT) as an objective comparator, and cardiologists-adjudicated AHF as reference standard. **Methods:** An observational study of 240 consecutive non-critical patients ≥50 years admitted with dyspnoea was conducted. Unstable AHF cases were deemed ineligible. Each modality was evaluated at the population level with area under the curve (AUC), sensitivity, and specificity, and compared at the patient level using conditional odds ratio for the association to AHF adjudicated by blinded cardiologists. Congestion was defined by LUS as (a) ≥3 B-lines bilaterally, or (b) B-lines combined with pleural effusion, and (c) CXR, interpreted by two thoracic radiologists, using (d) LDCT as an objective comparator. **Results:** Among 240 patients (66 with cardiologist-adjudicated AHF, 58 with chronic heart failure), LUS (b) demonstrated a diagnostic accuracy at population level of AUC = 0.82 (sensitivity = 80%, specificity = 84%), while CXR (c) achieved AUC = 0.80 (sensitivity = 68%, specificity = 91%), with CXR showing a modest but statistically significant difference over LUS at the patient level (OR = 1.51, *p* = 0.03). Incorporating pleural effusion into LUS increased its AUC from 0.67 to 0.82 (a vs. b, *p* < 0.001). The objective comparator, LDCT (d), achieved an AUC = 0.92 (sensitivity = 74%, specificity = 96%). In patients with chronic heart failure, LUS (b) and CXR (c) performed comparably (*p* = 0.87), whereas in those without chronic heart failure, CXR was superior (*p* = 0.04). **Conclusions:** In non-critical, diagnostically challenging patients with dyspnoea, in whom critical AHF cases were not eligible, including pleural effusion improved LUS accuracy for AHF. Diagnostic performance differed by heart failure history, with CXR superior in new-onset heart failure, while LUS and CXR performed comparably in chronic heart failure.

## 1. Introduction

Lung ultrasound (LUS) is a widely utilized diagnostic tool in patients with suspected pulmonary congestion due to acute heart failure (AHF) as it is fast, non-invasive, cheap, and widely available [1,2]. Although seemingly attractive in the acute setting, clinical equipoise remains, and LUS has not received strong, top-tier guideline recommendations. Substantial variability in prior LUS studies may relate to differences in applied AHF reference diagnoses [3,4], and variations in patient populations—some including patients with known heart failure [5] and others excluding pulmonary disease [6]. LUS protocols also differed between studies, with varying scanning methods and positivity criteria [7,8]. The most common approach, which requires ≥3 B-lines in two zones on each side (a total of ≥12 B-lines) may overlook subtle or milder pulmonary congestion, defined in some protocols as 5–14 B-lines [7]. Pleural effusion is present in 50–100% of patients with heart failure [1,8] but B-lines cannot be assessed in areas obscured by pleural effusion. Current ESC guidelines or LUS protocols provide no specific recommendations for the assessment of congestion in patients with pleural effusion. Another diagnostic pitfall of LUS is the interpretation of B-lines in the absence of an established heart failure diagnosis. B-lines indicate a non-specific increased lung density, that may also appear in pneumonia, interstitial pulmonary disease, and acute lung injury/acute respiratory distress syndrome [1].

A recent position statement suggests that LUS and CXR should be the initial imaging modalities within 1–2 h for patients with suspected AHF and if findings are inconclusive, chest CT may help differentiate AHF from other conditions [9]. As CT is impractical for routine use in acute settings, LDCT was employed as a research tool and objective comparator to detect subtle abnormalities. The optimal position of CT within the diagnostic hierarchy for patients with suspected AHF remains to be fully established, pending comparative accuracy data against other modalities.

Unlike previous multicentre or emergency-based LUS trials [10,11] our study addresses an underrecognized diagnostic challenge in acutely admitted, non-critical patients above the age of 50 years with dyspnoea, where overlapping cardiac and pulmonary features often make the diagnosis of acute heart failure uncertain [2]. In contrast to critically ill patients with more overt signs and symptoms of heart failure seen in previous LUS trials, this study includes individuals who present with milder symptoms, and imaging findings are therefore subtler and more difficult to interpret. This specific population has been largely underrepresented in prior LUS research, underscoring the novelty and clinical relevance of our study.

To date, there is a lack of prospective, same-day, head-to-head comparison of lung ultrasound and chest radiography (CXR) in this diagnostically challenging population. In this study, we compare the feasible, non-invasive methods LUS and CXR for identifying AHF in non-critical patients with acute dyspnoea, using low-dose CT (LDCT) as an objective comparator and cardiologist-adjudicated AHF as the reference standard. Secondary aims include evaluating whether a history of heart failure or inclusion of pleural effusion in the LUS assessment affects diagnostic performance.

## 2. Materials and Methods

### 2.1. Study Design

We conducted a prospective observational study at Copenhagen University Hospital–Bispebjerg & Frederiksberg, Denmark. The study was conducted in accordance with the Declaration of Helsinki, and the protocol was approved by the Danish National Committee on Health Research Ethics (H-17000869) on 1 August 2017. All patients provided written informed consent.

### 2.2. Population

**Figure 1:**
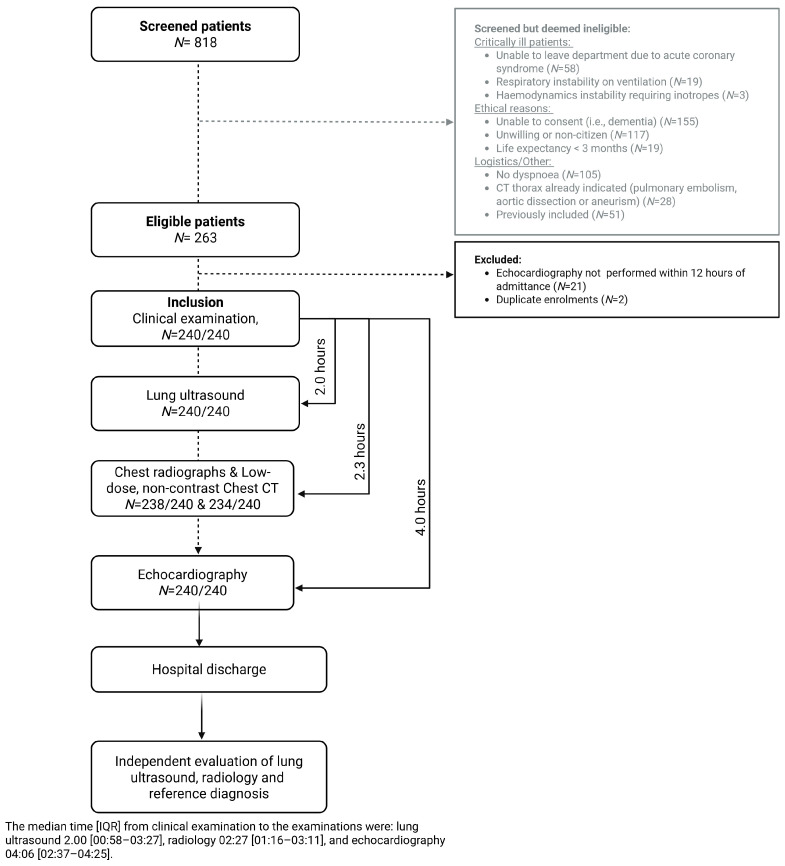
Flowchart of time to examinations.

We included adults (≥50 years) admitted to the emergency department and department of cardiology with acute dyspnoea as a primary or coprimary symptom, during 216 randomly selected weekdays, between November 2017 and August 2019 [12]. Protocolled study procedures were clinical examination, blood samples (including NT-proBNP), LUS, CXR, LDCT, and echocardiography (Figure 1). The main inclusion criterion was acute dyspnoea supported by one abnormal objective respiratory parameter (Table S1). The main exclusion criterion was absence of echocardiography within 12 h (Figure 1). For feasibility considerations during the ethical approval process, patients with respiratory or hemodynamic instability that precluded lying still for CT imaging were deemed ineligible.

Due to the eligibility criteria, all included patients were inherently non-critical. Operationally, “non-critical” was defined as the absence of respiratory or hemodynamic instability requiring intensive monitoring or acute interventions such as non-invasive ventilation, invasive ventilation, inotropic support, or continuous telemetry for suspected acute coronary syndrome. Patients were also required to be stable enough to undergo CT imaging safely. Consequently, the study cohort consisted predominantly of non-critical patients admitted for acute dyspnoea (Figure 1).

### 2.3. Lung Ultrasound

LUS was performed by certified examiners using a standardized 8-zone anterolateral protocol, complemented by 6 posterior zones to enhance the detection of pleural effusions [13]. Physicians performing LUS had completed a structured training program consisting of supervised scanning, theoretical instructions, standardized image acquisition protocols, and formal certification. LUS was performed using a cardiac probe with a lung preset and a scanning depth of 18 cm. A four-second clip was saved for all zones. The anterolateral zones were examined in a supine or semi-supine position. The six posterior zones were assessed in the upper, middle and lower thirds of the thorax.

Analysis of recorded LUS images were not performed bedside, but at a dedicated imaging setting by two certified, blinded readers, registering the number of B-lines and the presence of pleural effusion. LUS readers were blinded to all clinical information, echocardiography, and the radiologists’ interpretations. The highest number of B-lines in each zone was registered. A white lung pattern (confluent B-lines filling the entire screen) was counted as ten B-lines, while fused B-lines were quantified as the percentage of the screen divided by ten [14]. A positive zone was defined as ≥3 B-lines [14].

To evaluate various methods of assessing AHF on LUS, we defined a positive examination as follows:

Method (1) B-lines only: at least one positive zone on each hemithorax [10,15].

Method (2) Bilateral B-lines and/or pleural effusion: (a) method 1 criteria were met (at least one positive zone on each side) or (b) bilateral pleural effusion on lung ultrasound was present, regardless of B-line count.

Method (3) at least two positive zones per hemithorax [1,14].

Method (4) as method 3 and/or bilateral pleural effusion.

### 2.4. Radiology

Conventional CXR and LDCT were performed in continuation of each other. LDCT was included as a research tool to serve as an objective comparator. For patients unable to stand, CXR were performed in the supine position using an anteroposterior projection. The radiation dose of CXR was 0.1 mSV. The LDCT was performed with a low-dose protocol, without contrast, with a mean radiation dose of 1.3 mSV (1.2–1.4 mSV).

The CXR and LDCT were reviewed separately, by two specialized thoracic radiologists, blinded to all clinical information, study examinations, reference diagnosis, echocardiography, previous radiology and to the LUS readers’ interpretations. The radiologists evaluated cardiac and pulmonary pathology according to the Fleischner Society [16,17], with special focus on AHF [17,18,19]. The evaluation of radiological signs indicative of AHF was predefined and included ground-glass opacities, interlobar effusion, interlobular thickening, consolidation, atelectasis, crazy-paving, enlarged heart (anteroposterior projections), peribronchial cuffing, vascular redistribution (defined as increased vascular diameter/distention of the pulmonary veins), and pleural effusion [17,18,19]. For radiographs performed in supine position (33 patients, 14%), the same evaluation as for standing CXR were applied. However, interpretation accounted for known differences in the appearance of pulmonary congestion when supine, where vascular redistribution is less prominent. In these cases, greater weight was placed on signs such as fissural or septal thickening, pleural effusions, vascular indistinctness, and peribronchial cuffing. The probability of AHF was assessed on a 5-point Likert scale and agreement between the two radiologists on Likert item 4–5 was defined as AHF. Disagreements were deemed as no AHF.

### 2.5. Comprehensive Echocardiography

Echocardiography was performed and evaluated by cardiologists according to the ESC guidelines [20,21]. Cardiac dysfunction was defined as reduced left ventricular ejection fraction (LVEF) ≤ 40%, mildly reduced LVEF from 41 to 49%, preserved LVEF with diastolic dysfunction or heart failure due to severe valve disease. All echocardiographic examinations were subsequently reviewed and verified by at least one other cardiologist.

### 2.6. Clinical Reference Standard: Acute Heart Failure

We used two reference standards for AHF, as described in detail in Appendix A:

(1) Cardiologist-adjudicated AHF, adjudicated by two cardiologists. AHF was adjudicated based on the comprehensive echocardiography and medical record information, but without direct evaluation of radiology images or LUS. The AHF diagnosis was restricted to patients with AHF in the absence of concomitant clinically significant acute pulmonary disease. In sensitivity analyses presented in the Supplementary Material, we also report results for patients diagnosed with AHF who had concomitant significant acute pulmonary disease, as adjudicated by an expert panel of pulmonologists.
(2) A secondary, objective Echo-BNP AHF diagnosis. This was established to eliminate circular reasoning from medical record review that the radiology images might have influenced. It is based on four objective criteria: echocardiographic evidence of abnormal structure or function, elevated NT-proBNP, signs of increased left ventricular filling pressure, and treatment with loop diuretics.

A history of heart failure was determined based on a documented clinical diagnosis made by a cardiologist according to guideline-based criteria in the medical record.

### 2.7. Statistics

We used R version 4.2.2 for all statistical analyses. We report patient characteristics as means and standard deviations (SDs), medians and interquartile ranges (IQRs), or counts and percentages, as appropriate. Histograms and Shapiro–Wilks test of normality assessed distributions of variables. The independent samples *t*-test, the χ^2^-test/Fisher’s exact test, the Wilcoxon rank-sum, and the Kruskal–Wallis test were used for statistical comparisons. A *p*-value of <0.05 was considered significant, and all tests were two-sided.

Performance measurements at the population level for the association between the imaging modalities and AHF were demonstrated by sensitivity, specificity, negative predictive value (NPV), positive predictive value (PPV), positive likelihood ratio (PLR), and negative likelihood ratio (NLR). Diagnostic accuracy was assessed at two levels: at the group level for overall diagnostic performance using the area under the curve (AUC), and at the individual level for patient-level association with the diagnosis using conditional odds ratios. Throughout the manuscript, the term “odds ratio (OR)” refers specifically to the conditional odds ratio. The conditional odds ratio was stratified by patient ID, thereby accounting for paired or matched data in which each patient serves as their own control. This stratification mitigates bias from between-patient confounding factors that remain constant within an individual (e.g., underlying disease characteristics and interrater variability) and increases statistical efficiency by focusing on within-subject contrasts. The conditional odds ratio offers an advantage over AUC because it compares the two modalities within the same patients while holding other factors constant. AUC reflects group-level performance only. The metrics are not interchangeable, and the two metrics may diverge. Interrater reproducibility for LUS was assessed by independent, blinded re-evaluation of a subset of scans by a second experienced reader. Agreement for both LUS method 1 and method 2 was evaluated based on image interpretation, and interrater variability was quantified using the coefficient of variation and Cohen’s kappa statistics. For LDCT and CXR, two thoracic radiologists independently assessed both image quality and diagnostic findings in a similarly blinded manner. For research ethics approval, the required sample size during the first year was 98 to detect 85% sensitivity with a maximum 10% margin of error at the 95% confidence level [22]. Allowing for up to 10% missing data, a target of at least 110 patients was set. During the second-year enrolling, an additional 100 patients were recruited based on a prior power calculation requiring at least 90 participants [23]. Thus, a total sample size of 210 patients was expected. Parts of this dataset have previously been analyzed [12,24]. The current work extends those analyses by adding new statistical analyses and incorporating direct head-to-head comparisons of additional modalities.

## 3. Results

### 3.1. Baseline Characteristics

We enrolled 265 patients with acute dyspnoea. However, 2 patients had duplicate enrolments, and 21 patients were excluded because echocardiography was performed later than the pre-specified time window of 12 h, resulting in a final analytic sample of 240 patients. The number of screened patients deemed ineligible are summarized in Figure 1. The median time from admission until all study procedures were performed was 4 h (Figure 1). All patients underwent the standard anterolateral 8-zone ultrasound protocol, and 212 (88%) were also examined with the six posterior zones. CXR was available for 238 (99%), where 33 patients (14%) were examined in supine position. Due to logistical constraints within the radiology department, CT was performed in 234 patients (98%).

Cardiologist-adjudicated AHF, without clinically significant concomitant acute pulmonary disease, was observed in 66 patients (28%), and 81 (34%) patients met the criteria for the objective echo-BNP AHF diagnosis. Both diagnoses were identified in 63 patients (95%), indicating a strong agreement between the two definitions. In total, 58 patients (24%) had a history of chronic heart failure, and 35 patients (14.6%) have de novo heart failure. The patients with a history of heart failure had lower eGFR, more orthopnea, elevated levels of NT-proBNP at admission (Table 1), and significantly more abnormal echocardiographic findings than patients without history of heart failure (Table 1). Intravenous loop diuretics were administered to 11% (26/240) based solely on clinical examination, prior to LUS or any radiological imaging. Only one percent (3/240) received intravenous loop diuretics between LUS and radiology; these patients were still included in the analysis.

Bilateral pleural effusion was detected in 53/240 patients (22%), and any pleural effusion in 77/240 patients (32%). Among patients with cardiologists-adjudicated AHF, 67% (44/66) had bilateral pleural effusion on LUS, and 82% (54/66) had any type of pleural effusion on LUS. Of the 174 patients without AHF, 9 had bilateral pleural effusion (5%) and 23 (13%) had pleural effusion of any type.

### 3.2. Diagnostic Accuracy of LUS and Chest Radiography Using LDCT as Comparator

**Figure 2:**
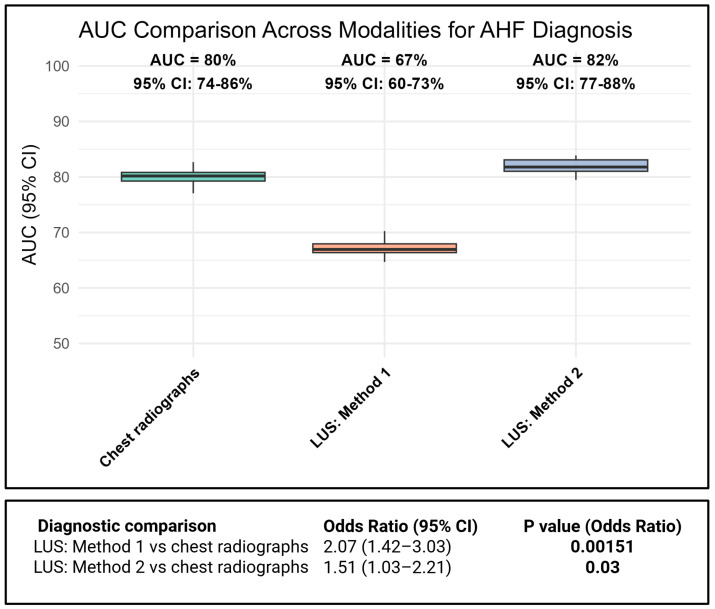
Comparison of AUC values at the group level and odds ratios at the patient level for the association between cardiologist-adjudicated AHF and different imaging modalities.

Both CXR and LUS demonstrated good diagnostic accuracy at the population level, as reflected by their AUCs for identifying cardiologist-adjudicated AHF, whereas CXR (AUC = 0.80 [0.74–0.86] significantly outperformed LUS method 1 (AUC = 0.67 [0.60–0.73]; OR = 2.07; 95% CI: 1.42–3.03; *p* = 0.002) and LUS method 2 (AUC = 0.82 [0.74–0.86]; OR = 1.51; 95% CI: 1.03–2.21; *p* = 0.03) at the patient level, as seen in Figure 2 (Figure S1). Including the assessment of pleural effusion in addition to B-lines significantly improved the diagnostic performance of LUS (AUC = 0.67 [0.60–0.73] vs. 0.82 [0.74–0.86]; *p* < 0.001). The objective comparator, LDCT, demonstrated an AUC of 0.92 [0.88–0.96] towards cardiologist-adjudicated AHF. Using echo-BNP AHF diagnosis as the reference instead of cardiologist-adjudicated AHF showed similar diagnostic performance (Figure S2), as did employing LUS methods 3 and 4, involving two or more zones bilaterally (Figure S3). Using AHF with concomitant acute pulmonary disease (*N* = 91) as the reference diagnosis, all methods showed declines in diagnostic performance. The corresponding AUCs were 0.74 for chest radiography, 0.64 for lung ultrasound without pleural effusion, and 0.75 for lung ultrasound with pleural effusion (Figure S4).

Including the assessment of pleural effusion notably improved the LUS sensitivity from 47% to 80%, while maintaining a specificity ≥84 (Table 2). For LUS method 2, 66% of positive results were correct, compared with 75% for CXR. When bilateral pleural effusion was included, the ability of LUS to correctly identify true cases improved, with an increase in PLR from 4.31 to 5.18 (Table 2). CXR performed even better, with a PLR of 7.91. As an objective comparator, LDCT correctly identified 88% of true cases (Table 2). Using the Echo-BNP AHF reference diagnosis demonstrated similar results (Table S2). In patients with AHF and concomitant acute pulmonary disease, all modalities showed reduced sensitivity, while specificities remained preserved (Table S3). In the exploratory analyses, LUS methods 3 and 4, requiring ≥two positive zones per hemithorax, demonstrated lower sensitivity but higher specificity (Table S4). The results of exploratory analyses are available in the Supplementary Material.

### 3.3. Relative Diagnostic Accuracy and Influence of Chronic Heart Failure

The distribution of heart failure phenotypes on echocardiography among patients with a history of heart failure was as follows: severe valvular disease 2%, HFpEF 17%, HFmrEF 26%, and HFrEF 52%. The remaining 3% showed no cardiac dysfunction of echocardiography.

In hypothesis-generating analyses for subgroups stratified by heart failure history, the AUCs were consistently, although not significant, higher across all modalities in patients without chronic heart failure compared to with those with chronic heart failure at the population level (LUS method 1: 68% vs. 61%, *p*: 0.379 and LUS method 2: 85% vs. 76%, *p*: 0.191). This was also demonstrated for the objective comparator, LDCT (94% vs. 91%, *p*: 0.525). The difference reached statistical significance only for CXR (AUC 84% vs. 70%, *p*: 0.0045).

**Figure 3:**
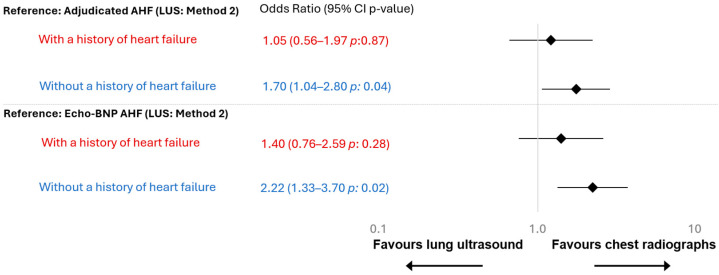
Odds ratio stratified by chronic heart failure. Heart failure history was based on documented clinical diagnoses in the medical record. In total, 58 patients (24%) had a chronic heart failure diagnosis, and 182 (76%) did not. Red indicates patients with a history of heart failure, and blue indicates those without.

At the individual-level comparison using conditional odds ratio, CXR and LUS method 2 performed comparably in patients with chronic heart failure (OR: 1.05 [0.56-1.97], *p*: 0.87, Figure 3). However, in patients without chronic heart failure, CXR showed a modest but statistically significant difference over LUS method 2 (OR: 1.70, 1.04–2,80, *p*: 0.04). These results were confirmed using Echo-BNP AHF as reference (*p*: 0.02), with even more pronounced results (Figure 3).

### 3.4. Interobserver Variability

The interobserver agreement for LUS was substantial, with κ: 0.66 (95% CI 0.43–0.84) for method 1 and κ: 0.74 (95% CI 0.57–0.89) for method 2. Agreement for CXR was similar to LUS method 2 (κ: 0.73, 95% CI 0.63–0.82). The highest agreement was observed for the objective comparator, LDCT (κ 0.88, 95% CI 0.81–0.95), as previously reported [12].

## 4. Discussion

### 4.1. Main Findings

This prospective observational study in non-critical emergency department patients with dyspnoea presents the first head-to-head comparison of lung ultrasound and CXR for detecting acute heart failure, using low-dose CT as an objective comparator and cardiologist adjudication as the reference standard. Our study has three main findings: First, incorporating bilateral pleural effusion substantially improved the diagnostic accuracy of LUS for detecting congestion in this diagnostically challenging, non-critical population. Second, CXR overall outperformed LUS, independent of reference diagnosis being cardiologist-adjudicated AHF or objective echo-BNP AHF. Third, in hypothesis-generating subgroup analyses of patients with chronic heart failure, LUS was as good as CXR, whereas in patients without a history of chronic heart failure, CXR appeared better than LUS, despite few patients with de novo heart failure.

### 4.2. LUS Versus Chest Radiographs

Although several systematic reviews and meta-analyses [3,25] have compared LUS and CXR for detecting AHF, our study adds evidence by focusing on non-critical emergency patients presenting with undifferentiated dyspnoea. This patient group is particularly relevant because imaging performance may differ when patients are clinically stable, symptoms are less overt, and multiple comorbidities (e.g., COPD, pneumonia, or renal dysfunction) may blur the distinction between cardiac and non-cardiac causes.

Previously reported sensitivities for CXR range from 14 to 76.5% [9,25,26], and for LUS from 40 to 97% for LUS [25,26,27], reflecting substantial variability across studies. In our study, CXR had a sensitivity of 68% and a specificity of 91%. In comparison, the sensitivity of LUS ranged from 47 to 80%, depending on methodology, while maintaining a high specificity (84–86%).

Previous studies reporting high sensitivities of LUS may differ by including cohorts with a higher pretest likelihood of AHF, excluding pulmonary diseases [28,29] or including younger individuals (>18 years) [6]. Such designs affect disease prevalence and likelihood, and the high sensitivities may not be generalizable to all acute cohorts, especially those with only subtle signs.

Sartini et al. compared LUS, CXR, and NT-proBNP against an expert panel AHF diagnosis, and although a subset of patients received diuretics prior to LUS, they reported that none of the tests alone provided sufficient diagnostic accuracy for AHF [26]. While untimely considerations may compromise the diagnostic accuracy of LUS, only 11% of patients in the current study received intravenous loop diuretics based on clinical examination alone, and just 1% between LUS and radiology. The low sensitivity of LUS in the current cohort most likely reflects a more subtle degree of pulmonary congestion in non-critical patients.

An important consideration for CXR is the patient’s ability to stand or sit. In our study, 33 CXRs (14%) were performed in the supine position, which can reduce diagnostic accuracy. Supine CXRs are known to underestimate pulmonary congestion and pleural effusion, as vascular redistribution is less apparent and effusions layer posteriorly, lowering sensitivity compared with upright projections. This should be considered when interpreting our CXR accuracy estimates, as CXR performance in this cohort is likely underestimated. In contrast, lung ultrasound is less affected by patient positioning; although dependent findings may be harder to detect in supine examinations, its overall diagnostic performance remains more robust than that of supine CXR.

### 4.3. The Optimal LUS Approach in the Acute Setting

Although LUS has demonstrated good diagnostic performance in AHF in some studies, its utility in non-critical emergency patients with undifferentiated dyspnoea is less established. Some studies characterized mild pulmonary edema as the presence of 5–14 B-lines [7]. However, the most widely used criterion, requiring ≥3 B-lines in two bilateral zones (a total of ≥12 B-lines), likely diagnose moderate to severe congestion, underrecognizing early-stage cases in more non-critical patients. We examined the effect of using one positive LUS zone bilaterally as a fast, simple method to also detect patients with AHF. A previous methodological study of patients in the emergency department showed that using one zone bilaterally improved both C-index value and sensitivity in patients with an unclear diagnosis of AHF [10]. We find that two zones on each hemithorax may be overly stringent, potentially overlooking patients with mild cases of AHF. This is reflected by the decrease in sensitivity (Table S3) and the lower AUC of LUS method 1 compared to method 2 (Figure 2).

As this was a single-center study, patient characteristics, referral patterns and local clinical routines may differ from those in other hospitals, which may affect the diagnostic performance. We sought to reduce technical variability through predefined imaging protocols, independent blinded readers and assessment of reproducibility, yet the results may still reflect the specific context of our institution. Validation in external populations using equally rigorous methods will therefore be important to confirm the robustness of these findings.

Most patients with AHF had pleural effusions (82%), often bilateral (67%). Because effusions can cause compression atelectasis and obscure B-lines, AHF should not be excluded based on absent B-lines. Especially in patients with a history of chronic heart failure, bilateral effusion documented by LUS is a strong supportive indicator of AHF. However, since pleural effusions may occur in other conditions such as malignancy, chronic lung disease, or nephrosis, LUS should not be the only imaging modality in patients with undifferentiated dyspnoea and no history of chronic heart failure.

In addition to these considerations, it is important to note a difference between the performance metrics used in our analyses. Although LUS method 2 had a slightly higher AUC than CXR, the conditional odds ratio favored CXR at the patient level. This reflects that the AUC captures overall population-level discrimination, whereas the conditional odds ratio evaluates which test is more often correct within the same patient. The conditional odds ratio may be particularly informative when several imaging modalities are assessed concurrently, as it reduces between-patient variability. Consequently, because these metrics measure different aspects of performance, they may not always align. The conditional odds ratio, which relies on paired data, is less commonly reported. By including both measures, we provide complementary insights into diagnostic performance that would not be captured by either metric alone.

### 4.4. Diagnostic Performance of LUS in Patients with and Without a History of Heart Failure

In our hypothesis-generating subgroup analyses, using conditional odds ratio at the patient level, chest radiograph and LUS were comparable for patients with chronic heart failure, but chest radiograph was significantly superior in patients without a history of chronic heart failure. This pattern likely reflects persistent structural and radiographic changes in patients with chronic heart failure, where both modalities similarly detect interstitial fluid. In contrast, CXR appears more informative in patients without a history of chronic heart failure. Although our results were significant, they should be interpreted with caution given the limited number of patients with de novo heart failure. As these analyses were hypothesis-generating, future multicenter research should examine whether diagnostic thresholds or image interpretation approaches should differ between de novo and chronic heart failure.

### 4.5. LDCT as Objective Comparator

Because chest CT is often considered the reference standard for pulmonary congestion, few studies have directly compared LUS, CXR and CT in diagnosing AHF, limiting evidence on how these bedside modalities perform relative to each other. LDCT was included as an objective comparator rather than a reference standard to avoid bias toward CXR, and it demonstrated the highest overall diagnostic accuracy. However, LUS with bilateral pleural effusion achieved a sensitivity of 80% and CXR 68%, compared with 74% for LDCT, supporting LUS as a rapid bedside triage method in the emergency setting.

We have previously shown in a pig model that CT detects subclinical congestion before B-lines appear on LUS, suggesting that LUS is less sensitive in early disease but remains a reliable indicator once congestion is present [30]. Siwik et al., similarly, found that redistribution in early pulmonary congestion causes no distinctive LUS changes but is detectable on both CXR and CT [2].

### 4.6. Strengths and Limitations

Our study has some limitations. First, the study was a single-centered study. Second, many patients were ineligible due to an inability to provide informed consent (e.g., patients with dementia), and these patients might be more difficult to diagnose due to a reduced ability to express symptoms. Third, 14% of CXRs were performed supine in less-mobile patients, which may introduce selection bias toward a sicker subgroup, limiting generalizability. Fourth, because CT was mandatory in every patient in this protocol, we included patients aged ≥50 years to minimize unnecessary radiation [31,32] and focus on a population with higher comorbidity prevalence [33]. While this limits generalizability, it reduces bias from enrolling healthier individuals with few comorbidities, who rarely pose the diagnostic challenge of AHF. Fifth, subgroup analyses stratified by history of heart failure should be regarded as hypothesis-generating only. Lastly, despite blinding the adjudicating cardiologists to radiology and LUS images, there may still be an overestimation of results due to potential influence on therapeutic management or written documentation in medical records.

Our study also had several strengths, offering unique insights in a challenging acute care setting. We prospectively enrolled a well-defined, non-critical emergency population of diagnostically challenging patients with dyspnoea, an understudied group compared with previous studies conducted in intensive care units or in high-risk heart failure settings, and conducted a same-day, blinded head-to-head comparison of LUS and CXR. Second, because the examinations were only four hours apart, any changes in fluid status during this short interval were minimal. This narrow timeframe enhances comparability between imaging methods and strengthens the reliability of the head-to-head assessment. Third, these findings are important since echocardiography is often unavailable the first hours of emergency admissions. Lastly, we used a standardized criterion to determine the final diagnosis of AHF, and results were confirmed with a secondary, observer-independent diagnosis.

## 5. Conclusions

In non-critical emergency, patients with acute dyspnoea, in whom critical AHF cases were ineligible, incorporating pleural effusion assessment substantially improved LUS sensitivity, resulting in diagnostic performance comparable to CXR in patients with known heart failure. In contrast, in these hypothesis-generating subgroup analyses stratified by heart failure history, CXR showed a modest but statistically significant advantage over LUS in patients without known heart failure.

### Clinical Applicability

LUS is supported as a first-line, radiation-free modality for detecting pulmonary congestion consistent with AHF in non-critically ill patients, particularly among those with a history of chronic heart failure, when bilateral pleural effusion and at least one positive zone per hemithorax are present. Its clinical use will depend on operator training and equipment availability, and CXR should be performed when LUS is unavailable or inconclusive, especially in patients without a history of heart failure, for whom CXR demonstrated modestly better performance. To balance diagnostic accuracy with radiation safety, LDCT should be reserved for selected cases where LUS and CXR remain inconclusive.

## Figures and Tables

**Table 1:** Patient characteristics of all included patients.

	All Patients	No History of HF	History of HF	*p*-Value
	*N*: 240	*N*: 182	*N*: 58	
**History**				
Age (years), mean (SD)	74.0 (10.2)	73.5 (10.5)	75.7 (9.06)	0.096
Sex (male), *N* (%)	133 (55.4)	94 (51.6)	39 (67.2)	0.054
Diabetes (I+II), *N* (%)	59 (24.6)	42 (23.1)	17 (29.3)	0.432
Hypercholesterolaemia, *N* (%)	71 (29.6)	43 (23.6)	28 (48.3)	**0.001**
Hypertension, *N* (%)	148 (61.7)	111 (61.0)	37 (63.8)	0.820
Kidney disease, *N* (%)	22 (9.17)	10 (5.46)	12 (20.3)	**0.001**
COPD, *N* (%)	126 (52.5)	102 (56.0)	24 (41.4)	0.072
**Clinical examination**				
BMI (kg/m^2^), median [IQR]	25.8 [22.7;29.9]	25.8 [22.9;29.4]	26.4 [22.3;30.6]	0.657
Systolic blood pressure (mmHg), mean (SD)	144 (27.8)	145 (27.7)	138 (27.5)	0.082
Respiratory frequency (*N*/minute), median [IQR]	22.0 [18.0;24.0]	22.0 [19.0;24.0]	20.5 [18.0;25.5]	0.633
Orthopnoea, *N* (%)	117 (48.8)	79 (43.4)	38 (65.5)	**0.005**
Pedal oedema, *N* (%)	72 (30.0)	52 (28.6)	20 (34.5)	0.490
Fever, *N* (%)	29 (12.1)	25 (13.7)	4 (6.90)	0.246
Cough, *N* (%)	180 (75.0)	140 (76.9)	40 (69.0)	0.296
Systolic murmur, *N* (%)	74 (30.8)	51 (28.0)	23 (39.7)	0.132
Bilateral rales, *N* (%)	89 (37.1)	62 (34.1)	27 (46.6)	0.119
Rhonchi, *N* (%)	76 (31.7)	65 (35.7)	11 (19.0)	**0.026**
Pao2/fio2 ratio, mean (SD)	320 (107)	317 (105)	330 (111)	0.431
NYHA class, *N* (%)				0.603
*II*	76 (31.7)	61 (33.5)	15 (25.9)	
*III*	105 (43.8)	76 (41.8)	29 (50.0)	
*IV*	58 (24.2)	44 (24.2)	14 (24.1)	
NT-proBNP (pg/mL), median [IQR]	867 [228;3209]	599 [169;2385]	2694 [814;6008]	**<0.001**
C-reactive protein (mg/L), median [IQR]	16.9 [5.88;61.7]	15.2 [5.06;61.0]	21.0 [7.25;61.3]	0.316
eGFR (mL/min/1.73 m^2^), median [IQR]	69.5 [49.0;85.0]	75.0 [52.0;88.9]	54.5 [42.2;70.0]	**<0.001**
**Echocardiography**				
LVEF (%), median [IQR]	55.0 [45.0;60.0]	60.0 [50.0;60.0]	40.0 [25.0;50.0]	**<0.001**
TR velocity (m/s), mean (SD)	275 (74.4)	272 (76.6)	284 (67.0)	0.269
Average E/e, median [IQR]	10.6 [7.90;14.5]	10.3 [7.57;13.4]	12.4 [8.30;18.5]	**0.007**
LA-volume index (mL/m^2^), mean (SD)	34.2 (15.5)	31.9 (14.7)	41.6 (15.8)	**<0.001**
Increased filling pressure (II+III), *N* (%)	94 (39.2)	59 (32.4)	35 (60.3)	**<0.001**
Echocardiographic cardiac function, *N* (%)				
*Severe valvular heart disease*	9 (3.75)	8 (4.40)	1 (1.72)	0.691
*LVEF ≤ 40*	54 (22.5)	24 (13.2)	30 (51.7)	**<0.001**
*LVEF 41–49*	24 (10.0)	9 (4.95)	15 (25.9)	**<0.001**
*LVEF > 50 + (definite diastolic dysfunction or LAE/LVH)*	48 (20.0)	38 (20.9)	10 (17.2)	0.678

Bold for *p*-values indicates significant differences.

**Table 2:** Diagnostic accuracy table for all included patients using Clinical Reference AHF, adjudicated by cardiologists, as reference.

DIAGNOSTIC MODALITY	Sensitivity (%)	Specificity (%)	PPV (%)	NPV (%)	PLR	NLR
	(95% CI)	(95% CI)	(95% CI)	(95% CI)	(95% CI)	(95% CI)
**LUNG ULTRASOUND**						
Method 1	47	86	56	81	3.41	0.62
(≥3 B-lines in one zone bilaterally)	(35–60)	(80–91)	(42–70)	(75–86)	(2.17–5.35)	(0.49–0.78)
Method 2	80	84	66	92	5.18	0.23
(≥3 B-lines in one zone bilaterally and/or bilateral PE)	(69–89)	(78–90)	(55–76)	(87–96)	(3.59-7.47)	(0.14–0.38)
**RADIOLOGY**						
Chest radiography	68	91	75	88	7.91	0.35
	(56–79)	(86–95)	(62–85)	(83–93)	(4.74–13.18)	(0.24–0.50)
LDCT	74	96	88	91	18.45	0.27
	(62–84)	(92–98)	(76–95)	(86–95)	(8.81–38.66)	(0.18–0.40)

PPV: positive predictive value, NPV: negative predictive value, PE: pleural effusion, PLR: positive likelihood ratio, NLR: negative likelihood ratio, LUS Method 1: ≥one positive zone bilaterally, LUS Method 2: ≥one positive zone bilaterally and/or bilateral pleural effusion, Radiology: agreement between the two radiologists on pulmonary congestion.

## Data Availability

The datasets presented in this study are not publicly available due to ethical restrictions associated with patient confidentiality. According to the approval granted by the Institutional Review Board, individual-level data cannot be shared outside the research team.

## Supplementary Materials

The following supporting information can be downloaded at https://www.mdpi.com/article/10.3390/diagnostics15233047/s1, Figure S1: AUC comparison at the group level across all imaging modalities and LUS methods for the Clinical Reference AHF diagnosis. *p*-values provided are from comparisons at the group level between AUCs using DeLong Test. Table S1: Table of abnormal objective respiratory parameters considered to support acute dyspnoea at patient inclusion; Figure S2: Conditional odds ratio for the association between Echo-BNP AHF as reference diagnosis and the imaging modalities. Table S2: Diagnostic accuracy table for all included patients, using the secondary reference diagnosis (Echo-BNP AHF) as the reference; Figure S3: Conditional odds ratio for the association for LUS in relation to Clinical Reference AHF as reference diagnosis and the different imaging modalities for method 1–4. Table S3: Diagnostic accuracy table for all included patients, using the combination of strict AHF and concomitant significant acute pulmonary disease, as adjudicated by pulmonologists, as the reference diagnosis; Figure S4: AUC comparison at the group level for AHF with concomitant acute pulmonary disease (*N* = 91); Table S4: Diagnostic accuracy table for all included patients for method 1–4 using Clinical Reference AHF, adjudicated by the cardiologists, as reference.

## Abbreviations

The following abbreviations are used in this manuscript:

AHF	Acute heart failure
AUC	Area Under the Receiver Operating Characteristic Curve
OR	Conditional Odds Ratio
CT	Computed Tomography
CXR	Chest Radiography (Chest X-ray)
HF	Heart Failure
IQR	Interquartile Range
LDCT	Low-dose, Non-contrast Chest Computed Tomography
LUS	Lung Ultrasound
LVEF	Left Ventricular Ejection Fraction
NLR	Negative Likelihood Ratio
NPV	Negative Predictive Value
NT-proBNP	NT-proBrain Natriuretic Peptide
NYHA	New York Heart Association Functional Classification
PLR	Positive Likelihood Ratio
PPV	Positive Predictive Value
ROC	Receiver Operating Characteristic Curve
SD	Standard Deviation
TNR	True Negative Rate
TPR	True Positive Rate

## Appendix A. Overview of the Reference Standards for AHF

(1) **Clinical Reference AHF**: Adjudicated by two cardiologists (and a third in case of disagreement) according to a modified version of the 2017 cardiovascular and stroke endpoint definitions for clinical trials consensus report [12,20,21,34,35] based on the following information:
  (A) Review of comprehensive echocardiography images with evidence of abnormal structure, function, and LV filling pressures (grade II + III)
  (B) Review of medical record information including history and blood samples, but without direct evaluation of radiology images.
  (C) Review of the presence of clinically significant concomitant acute pulmonary disease as a potential cause of acute dyspnea

The cardiologists were blinded to LUS, CT, and chest radiograph images.
**AHF was adjudicated by two cardiologists using a 5-point Likert scale, where Likert 5 was considered as Clinical Reference AHF.**

(2) **Echo-BNP AHF:** An operator independent objective diagnosis established to eliminate any bias and circular reasoning cardiologists would obtain by reviewing the medical record review that the radiology imaging modalities might have influenced.
  Based only on **the presence of all four objective criteria**:
  (1) Echocardiographic abnormal structure or function; left ventricular ejection fraction (LVEF) ≤ 40%, LVEF 41–49%, LVEF ≥ 50% with diastolic dysfunction or severe valve disease [34];
  (2) NT-proBNP >300 pg/mL [34];
  (3) Signs of elevated LV filling pressure on echocardiography (grade II + III) [21];
  (4) Administration of loop diuretics orally or intravenously any time during admission or at dis-charge
