# Supplementary material for: Lung Ultrasound Versus Chest Radiography for Acute Heart Failure: Impact of Heart Failure History and Pleural Effusion
Source: Diagnostics (Basel). 2025 Nov 28;15(23):3047. doi: 10.3390/diagnostics15233047 (PMC12691748; doi:10.3390/diagnostics15233047)
Supplement: Supplementary file 1 [file diagnostics-15-03047-s001.zip › diagnostics-3994888-supplementary.pdf]

Supplementary material

Supplementary Table S1 2
Supplementary Table S2 3
Supplementary Table S3 4
Supplementary Table S4 5
Supplementary Figure S1 6
Supplementary Figure S2 8
Supplementary Figure S4 9
Supplementary Figure S4 6

Supplementary Table S1: Table of abnormal objective respiratory parameters considered to support acute dyspnoea at patient inclusion.

**Objective parameters supporting respiratory imbalance:**
Respiratory rate >20 breaths/minute
Saturation ≤95% (≤92% if chronic obstructive pulmonary disease)
Abnormal pO2 or pCO2 in arterial blood
Objective signs of heart failure (i.e. jugular vein distention, peripheral oedema, orthopnoea, or bilateral rales on auscultation)
Rhonchi or prolonged breathing on auscultation

Supplementary Table S2: Diagnostic accuracy table for all included patients, using the secondary reference diagnosis (Echo-BNP AHF) as the reference.

	Sensitivity (%) (95% CI)	Specificity (%) (95% CI)	PPV (%) (95% CI)	NPV (%) (95% CI)	PLR (95% CI)	NLR (95% CI)
**LUNG ULTRASOUND**						
Method 1	44	88	65	76	3.72	0.63
(≥3 B-lines in one zone bilaterally)	(33-56)	(82-93)	(51-78)	(69-82)	(2.29-6.05)	(0.52-0.77)
Method 2	69	85	70	84	4.58	0.36
(≥3 B-lines in one zone bilaterally and/or bilateral PE)	(58-79)	(78-90)	(59-80)	(78-90)	(3.08-6.81)	(0.26-0.51)
Bilateral pleural effusion	53	94	83	80	9.38	0.50
	(42-64)	(90-97)	(70-92)	(73-85)	(4.81-18.27)	(0.39-0.63)
**RADIOLOGY**						
Chest radiography	58	92	0.78	81	7.10	0.46
	(47–69)	(86–96)	(66–88)	(75-87)	(4.08–12.34)	(0.35-0.59)
LDCT	62	97	91	83	19.90	0.39
	(51–73)	(93–99)	(80–97)	(77–88)	(8.26–47.94)	(0.30-0.52)

PPV: positive predictive value, NPV: negative predictive value, PLR: positive likelihood ratio, NLR: negative likeli-hood ratio, LUS Method 1: ≥one positive zone bilaterally, LUS Method 2: ≥one positive zone bilaterally and/or bilateral pleural effusion, Radiology: agreement between the two radiologists on pulmonary congestion

Supplementary Table S3: Diagnostic accuracy table for all included patients, using the combination of strict AHF and AHF and concomitant significant acute pulmonary disease, as adjudicated by pulmonologists, as the reference diagnosis.

**DIAGNOSTIC MODALITY**	**Sensitivity (%) (95% CI)**	**Specificity (%) (95% CI)**	**PPV (%) (95% CI)**	**NPV (%) (95% CI)**	**PLR (95% CI)**	**NLR (95% CI)**
**LUNG ULTRASOUND**						
Method 1 (≥3 B-lines in one zone bilaterally)	43 (33-54)	89 (83-94)	71 (57-82)	72 (65-79)	4.06 (2.42-6.83)	0.63 (0.3-0.77)
Method 2 (≥3 B-lines in one zone bilaterally and/or bilateral PE)	69 (58-78)	88 (82-93)	78 (67-86)	82 (79-88)	5.74 (3.64-9.05)	0.35 (0.26-0.48)
Bilateral pleural effusion	54 (0.44-0.65)	98 (94-100)	94 (84-99)	78 (72-84)	27.22 (8.74-84.78)	0.46 (0.37-0.58)
**RADIOLOGY**						
Chest radiographs	59 (48-69)	95 (91-98)	88 (77-95)	79 (73-85)	12.62 (6.00-26.54)	0.43 (0.34-0.55)
LDCT	60 (49-70)	99 (95-100)	96 (88-100)	80 (74-86)	45.00 (11.24-180.11)	0.41 (0.31-0.52)

PPV: positive predictive value, NPV: negative predictive value, PE: pleural effusion, PLR: positive likelihood ratio,
NLR: negative likelihood ratio, LUS Method 1: ≥one positive zone bilaterally, LUS Method 2: ≥one positive zone bilaterally
and/or bilateral pleural effusion, Radiology: agreement between the two radiologists on pulmonary congestion

Supplementary Table S4: Diagnostic accuracy table for all included patients for method 1-4 using Clinical Reference AHF, adjudicated by the cardiologists, as reference.

	Sensitivity (%) (95% CI)	Specificity (%) (95% CI)	PPV (%) (95% CI)	NPV (%) (95% CI)	PLR (95% CI)	NLR (95% CI)
**LUNG ULTRASOUND**						
Method 1 (≥3 B-lines in one zone bi-laterally)	47 (35-60)	86 (80-91)	56 (42-70)	81 (75-86)	3.41 (2.17, 5.35)	0.62 (0.49, 0.78)
Method 2 (≥3 B-lines in one zone bi-laterally and/or bilateral PE)	80 (69-89)	84 (78- 90)	66 (55-76)	92 (87-96)	5.18 (3.59- 7.47)	0.23 (0.14- 0.38)
Method 3 (≥3 B-lines in two zones bi-laterally)	26 (16-38)	95 (90-98)	65 (44-83)	77 (71-83)	4.98 (2.34-10.61)	0.78 (0.68-0.91)
Method 4 (≥3 B-lines in two zones bi-laterally and/or bilateral pleural effusion)	71 (59-82)	92 (87-96)	77 (65-87)	89 (84-93)	8.85 (5.23-14.26)	0.31 (0.21-0.46)
Bilateral pleural effusion	65 (52-76)	95 (90-98)	83 (70-92)	88 (82-92)	12.60 (6.51-24.38)	0.37 (0.26-0.51)

PPV: positive predictive value, NPV: negative predictive value, PLR: positive likelihood ratio, NLR: negative likelihood ratio, LUS Method 1: ≥one positive zone bilaterally, LUS Method 2: ≥one positive zone bilaterally and/or bilateral pleural effusion, LUS Method 3: ≥two positive zone bilaterally, LUS Method 4: ≥two positive zone bilaterally and/or bilateral pleural effusion

Supplementary Figure S1: AUC comparison at group level across all imaging modalities and LUS methods for the Clinical Reference AHF diagnosis. P-values provided are from comparisons at group level between AUCs using DeLong Test.

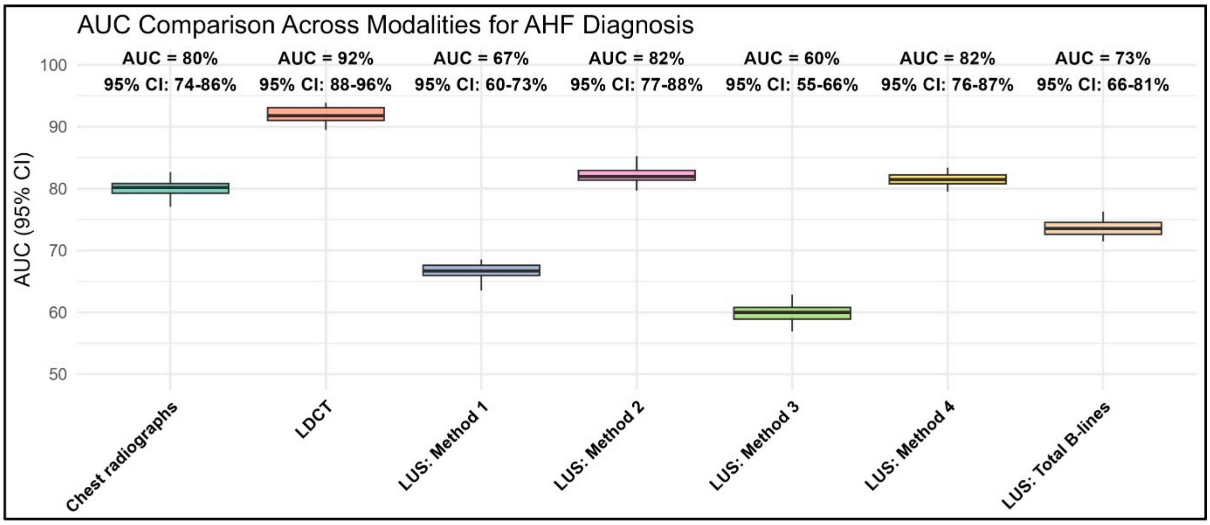

Modality 1	Modality 2	p-value
LUS: Method 1	Chest radiographs	0.00398
LUS: Method 1	LDCT	< 0.001
LUS: Method 1	LUS: Method 2	< 0.001
LUS: Method 1	LUS: Method 3	0.153
LUS: Method 1	LUS: Method 4	< 0.001
LUS: Method 1	LUS: Method 4	< 0.001
LUS: Method 2	LDCT	0.00459
LUS: Method 2	LUS: Method 3	< 0.001
LUS: Method 2	LUS: Method 4	0.844
LUS: Method 2	LUS: Total B-lines	0.0501
LUS: Method 3	LDCT	< 0.001
LUS: Method 3	LUS: Method 4	< 0.001
LUS: Method 3	LUS: Total B-lines	< 0.001
LUS: Method 4	LDCT	< 0.001
LUS: Method 4	LUS: Total B-lines	0.0331
LUS: Total B-lines	LDCT	< 0.001
Chest radiographs	LDCT	< 0.001
Chest radiographs	LUS: Method 2	0.532
Chest radiographs	LUS: Method 3	< 0.001
Chest radiographs	LUS: Method 4	0.634
Chest radiographs	LUS: Total B-lines	0.115

Supplementary Figure S2: Conditional odds ratio for the association between Echo-BNP AHF as reference diagnosis and the imaging modalities.

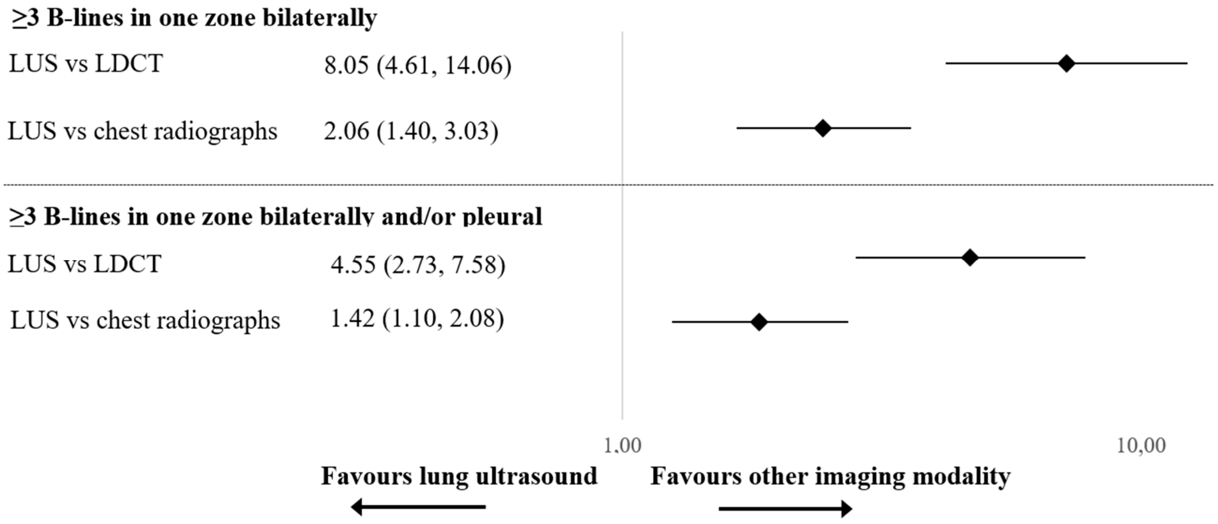

<u>Supplementary Figure S3:</u> Conditional odds ratio for the association for LUS in relation to Clinical Reference AHF as reference diagnosis and the different imaging modalities for method 1-4.

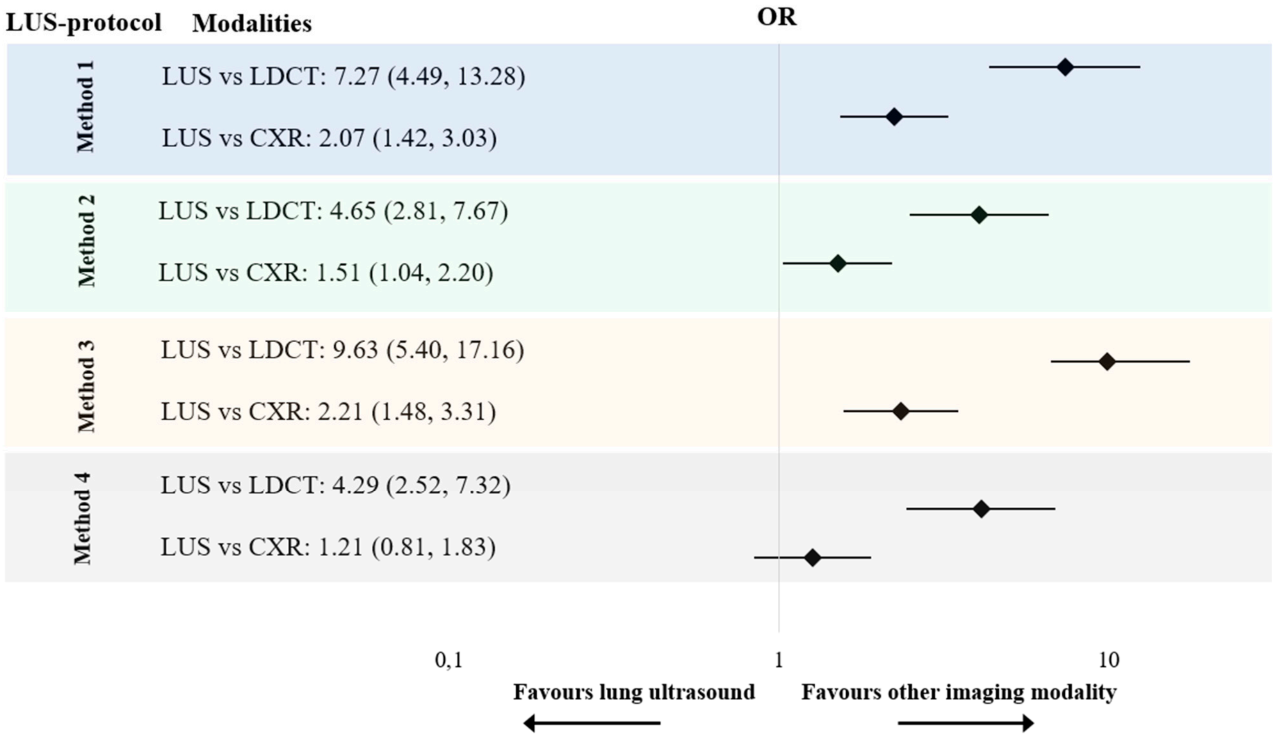

Supplementary Figure S4: AUC comparison at group level for AHF with concomitant acute pulmonary disease (N=91).

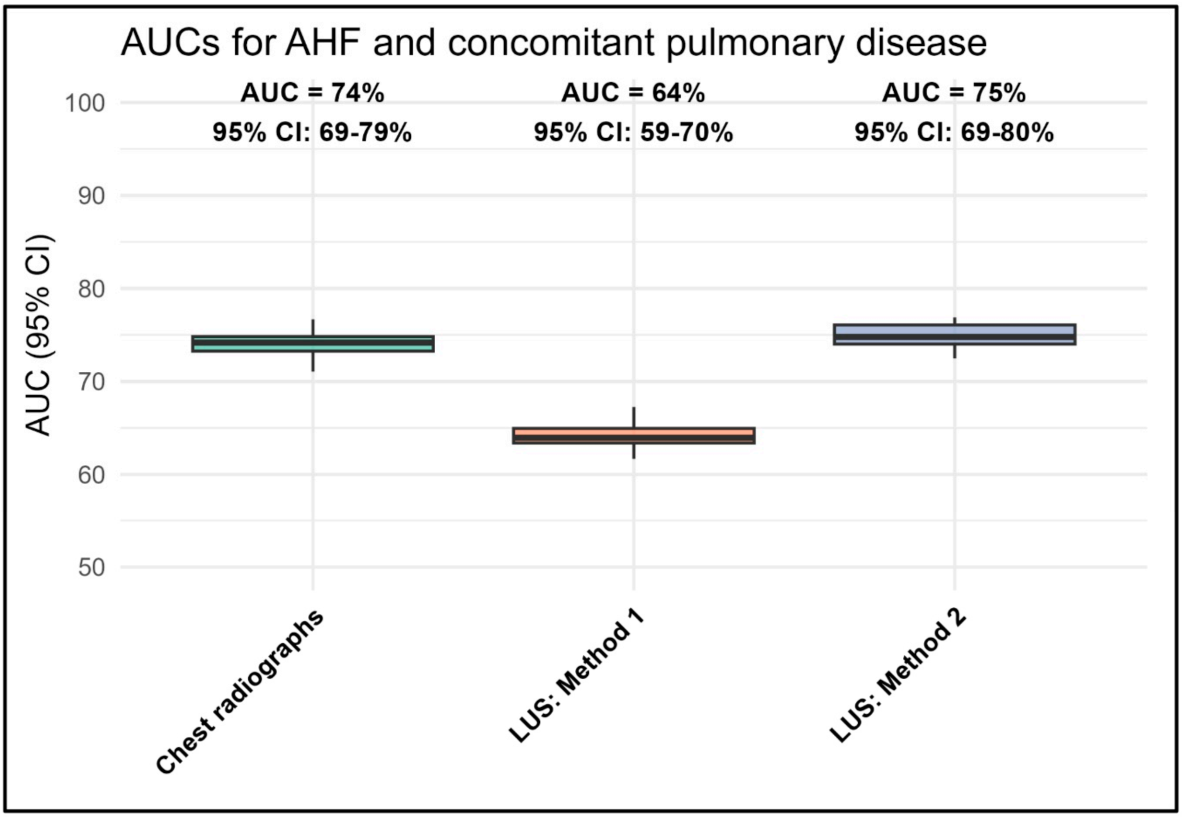

Diagnostic comparison	Odds Ratio (95% CI)	P value (Odds Ratio)
LUS: Method 1 vs chest radiographs	3.63 (2.42-5.45)	**<0.01**
LUS: Method 2 vs chest radiographs	2.35 (1.57-3.51)	**<0.01**
